# Impact of a community-led intervention on the uptake of childhood vaccines in Liverpool: a protocol for a synthetic control evaluation

**DOI:** 10.1136/bmjopen-2025-111500

**Published:** 2026-01-21

**Authors:** Mohammed Sherif Amin, Xingna Zhang, Mark Alan Green, Dawn Holford, Charlotte Hemingway, Amina Ismail, James Moran, Vicki Doyle, Cait Taylor, Miriam Taegtmeyer, Daniel Hungerford

**Affiliations:** 1 Department of Clinical Infection, Microbiology and Immunology, University of Liverpool, Liverpool, England, UK; 2 Department of Public Health and Policy, University of Liverpool, Liverpool, England, UK; 3 Health Protection Research Unit in Gastrointestinal Infections, University of Liverpool, National Institute for Health and Care Research, Liverpool, England, UK; 4 Department of Geography and Planning, University of Liverpool, Liverpool, England, UK; 5 School of Psychological Science, University of Bristol, Bristol, England, UK; 6 Department of International Public Health, Liverpool School of Tropical Medicine, Liverpool, England, UK; 7 Capacity Development International, Liverpool, England, UK; 8 Central Liverpool Primary Care Trust, Liverpool, England, UK; 9 Dingle Park Practice, Liverpool, England, UK; 10 Unit in Emerging and Zoonotic Infections, University of Liverpool, National Institute for Health and Care Research, Liverpool, England, UK

**Keywords:** Vaccination, PAEDIATRICS, EPIDEMIOLOGY, Public health, Primary Prevention

## Abstract

**Abstract:**

**Introduction:**

Vaccines are our best defence against infectious diseases, yet uptake of childhood immunisation programmes has consistently declined in the UK, with growing concerns around socioeconomic inequalities. Liverpool, in particular, demonstrated some of the lowest uptake rates in England since 2019. In response, the Health Equity Liverpool Project (HELP) implemented a hyper-localised community-led initiative between September 2023 and June 2024 to tackle vaccine hesitancy. Activities included outreach events and school-based engagement across nine sites within Liverpool. Despite promising qualitative evidence, the intervention’s impact on childhood vaccine uptake has not yet been quantified. We aim to evaluate the population level impact of the HELP intervention on the uptake of five childhood vaccines (first and second doses of the measles, mumps and rubella vaccine (MMR1, MMR2), 6-in-1 vaccine (diphtheria, tetanus, pertussis, polio, haemophilus influenzae type b and hepatitis B), pneumococcal conjugate vaccine booster dose (PCV) and rotavirus vaccine) using synthetic control methods.

**Methods and analysis:**

We will analyse publicly available quarterly vaccine uptake data (between April 2019 and March 2025) from the Cover of Vaccination Evaluated Rapidly programme for general practices (GPs) in England. The intervention group will be defined as practices located within a 1 km radius of the intervention sites. A synthetic control group will be constructed using non-intervention GPs matched on pre-intervention vaccine uptake, and linked demographic, socioeconomic and healthcare capacity covariates. Primary outcomes are the uptake of MMR1 and MMR2 vaccines. Secondary outcomes include the uptake of 6-in-1, PCV and rotavirus vaccines. Average treatment effects will be estimated as the post-intervention difference in uptake between intervention and synthetic control groups. Sensitivity analyses will examine spillover effects, alternative spatial definitions of exposure, the biasing effect of concurrent interventions and the feasibility of analysis at small area neighbourhood level.

**Ethics and dissemination:**

This study will be conducted as part of the ReCITE project, which has received ethical approval from the Liverpool School of Tropical Medicine Research Ethics Committee (Reference: 24–018) and is funded by the UK Arts and Humanities Research Council (Project Number: AH/Z505341/1). Findings will be shared with the project funder and submitted for publication in a peer-reviewed journal.

**STRENGTHS AND LIMITATIONS OF THIS STUDY**

- To our best knowledge, this will be among the first UK-based studies to use synthetic control methods for evaluating the impact of community-led intervention on childhood vaccine uptake.
- The Cover of Vaccination Evaluated Rapidly dataset used will provide a near universal coverage of children registered with a general practice in England, offering a large pool for constructing a robust synthetic control group.
- The study is sustainable, providing methodological transparency through plans to publish analytic code and any derived data, enabling replication and adaptation for future evaluation of similar community-led interventions across England.
- Despite our attempt to include a large set of covariates, there remains a potential for residual confounding from unobserved characteristics, such as parental vaccine attitudes, language barriers and undocumented outreach.
- The absence of a UK registry for reporting local childhood vaccination initiatives may limit our ability to identify other concurrent interventions affecting vaccine uptake in the control practices, potentially affecting the quality of matching and impact estimation.

## Introduction

 Vaccines are powerful public health tools, significantly reducing illnesses and mortality associated with vaccine-preventable diseases.^1 2^ Childhood vaccinations are especially valuable, as timely immunisation contributes to herd immunity,^3^ limiting the spread of infectious diseases within communities.

Despite their known effectiveness,^4^ coverage of several childhood vaccines has declined worldwide,^5^ falling below the WHO’s 95% recommended threshold.^6^ The UK in particular has experienced a steady decline in the uptake of all childhood vaccines since 2013.^7^ In 2023/2024, coverage in England for the first and second doses of the measles, mumps and rubella vaccine (MMR1 at age 2 years and MMR2 at age 5 years) was 88.9% and 83.9%, respectively.^8^ Liverpool figures were even lower during the same period, with just 81% for MMR1 at age 2 and 73.4% for MMR2 at age 5,^9^ placing the city among the worst performing areas nationally. This has resulted in several outbreaks across England,^10 11^ including a recent cluster of measles cases in Liverpool.^12^

Further compounding this issue are the persistent demographic and socioeconomic inequalities in childhood vaccines uptake.^13^,^16^ Children from deprived communities have notably lower vaccination rates than their peers in more affluent areas, with gaps widening over time.^14^ For instance, the absolute inequality in the uptake of MMR2 between the most and least deprived areas in England widened from 5.3% in 2019 to 11.5% in 2023.^14^

Several factors have contributed to these low and unequal uptake rates, with vaccine hesitancy being among the primary drivers (a delay or refusal to vaccinate despite the availability of vaccines^17^). Hesitancy is often associated with mistrust in healthcare systems, limited engagement with trusted local actors, inadequate communication from healthcare professionals, misinformation and disinformation (deliberate and targeted spreading of misinformation).^18 19^ These factors may intersect with structural barriers, such as inflexible appointment systems, further discouraging the uptake.^19^

A broad range of interventions has previously targeted childhood vaccination uptake, including reminder systems, educational campaigns, financial incentives and community engagement activities.^20^,^22^ A recent systematic review^21^ reported reminder/recall systems improve uptake by around 15%, education-based initiatives by 19% and financial incentives by up to 67%. Furthermore, evidence suggests multicomponent interventions, especially those built on dialogue, tailored to local contexts and involving engagement with community-trusted actors, are more effective in addressing vaccine hesitancy.^23^ This was highlighted during the COVID-19 vaccine rollout, where initiatives such as Community Champions were introduced nationally to promote uptake among marginalised populations.^24^

Incorporating these values into its approach, the Health Equity Liverpool Project (HELP) was established in 2021 to address persistent health inequities within deprived communities across Liverpool.^25^ One of its core aims was to improve childhood vaccine uptake in areas with persistent low coverage using community-led creative health methods, building on its experience of tackling health inequities in the Global South.^25^ Although the MMR vaccine was the primary focus, HELP adopted a broader strategy to tackle underlying barriers to vaccine hesitancy using the 5Cs framework (confidence, complacency, constraints, risk calculation and collective responsibility).^26^ This included hyper-localised outreach activities and school-based engagement sessions, which created valuable opportunities for parents and carers to engage in conversations with trusted individuals (general practitioners, nurses and community champions). A qualitative evaluation of the programme highlighted increased community conversations about childhood vaccines and improved engagement among parents and caregivers.^25^ Despite this promising evidence, a measurable impact on childhood vaccine uptake has not yet been assessed.

Quantitatively evaluating the impact of such hyper-localised community-led programmes represents a methodological challenge. While randomised controlled trials remain the gold standard, they are often unfeasible for evaluating complex health system interventions involving numerous interacting components and stakeholders within a dynamic social context. Routine healthcare data present an important opportunity to assess such initiatives at the population level. However, without appropriate control groups, it is difficult to attribute causal effects through simple pre–post comparisons. Quasi-experimental methods, such as synthetic control methods (SCM), offer a robust analytical alternative.^27 28^ SCM involves the construction of a group matched in characteristics to the intervention group (known as the ‘synthetic control’). This allows a counterfactual scenario to be estimated by replicating the pre-intervention trend in the outcome observed in the intervention group. In other words, the synthetic control demonstrates what would have happened in the absence of the intervention.^28^

We aim to apply the SCM to evaluate the impact of the HELP project on the uptake of five childhood vaccines, including MMR1 at ages 2 and 5, MMR2, the 6-in-1 vaccine (diphtheria, tetanus, pertussis, polio, haemophilus influenzae type b (Hib) and hepatitis B), pneumococcal conjugate vaccine booster dose (PCV) and rotavirus vaccine.

## Methods and analysis

### Data sources and measures

#### Vaccine uptake

This study will use publicly available data from the Cover of Vaccination Evaluated Rapidly (COVER) programme,^29^ which provides quarterly statistics on the uptake of routine childhood immunisations for children aged 5 years and under at the general practice (GP) level in England.

Our primary outcome is the uptake of the first and second doses of MMR vaccine (MMR1 and MMR2). We will further examine the 6-in-1 vaccine, PCV booster dose and rotavirus vaccine as secondary outcomes, as they are administered at similar ages and often coincide with MMR. Table 1 provides information on the age at which each vaccine is administered, based on the UK childhood vaccination schedule,^30^ and its corresponding COVER reporting age.

**Table 1:** Childhood vaccines included in the analysis, with their recommended administration ages and corresponding measurement points in the COVER dataset

Vaccine	Age administered	Age measured in COVER
DTaP/IPV/Hib/ HepB (6-in-1)	8, 12, 16 weeks	12 months
Rotavirus	8, 12 weeks	12 months
PCV booster	13 months	2 years
MMR1	13 months	2 years, 5 years
MMR2	3 years 4 months	5 years

COVER, Cover of Vaccination Evaluated Rapidly; DTaP, diphtheria, tetanus, pertussis; HepB, hepatitis B; Hib, haemophilus influenzae type B; IPV, inactivated polio vaccine; MenC, meningococcal group C; MMR, measles, mumps and rubella; PCV, pneumococcal conjugate vaccine.

COVER reports vaccine uptake as the percentage of eligible children who received the vaccine by a certain age within a given quarter. A key strength of this dataset is its completeness, with a near universal coverage across the GP-registered child population in England. We will use data spanning 24 consecutive quarters, from April 2019 (the earliest quarter with GP-level data) to March 2025, labelled sequentially from Quarter 1 (Q1) to Quarter 24 (Q24).

### Covariates

To account for factors that may influence the uptake of the childhood vaccines, we will incorporate a set of covariates that reflect the demographic, socioeconomic context and health-seeking behaviour of the GP-registered population, as well as a range of structural and financial characteristics of the practices. Covariates were chosen based on their theoretical relevance and supporting evidence from existing literature.

The number of children aged 0–4 years and the proportion of males will be included to account for differences in the size and sex structure of the child population in each practice. Socioeconomic deprivation^14 15^ and ethnicity^16^ have been consistently associated with lower immunisation coverage in the UK, possibly due to language barriers, misinformation and mistrust in health services.^31^ We will therefore include the 2019 Index of Multiple Deprivation (IMD) and the proportion of black, Asian and mixed ethnicity as reported in the 2021 census.

Healthcare capacity and quality are also critical, as they directly influence the accessibility and delivery of preventive services.^31^ We will use the percentage of Quality and Outcomes Framework achieved, clinical workforce per 10 000 patients, total funding per patient and average number of appointments per registered patient as proxies for institutional capacity and service availability. The proportion of patient-reported satisfaction with GP services and the proportion of those with caring responsibilities will also be included. These measures capture broader aspects of patient engagement^32^ and competing priorities,^33^ which can shape parents’ ability or willingness to access routine immunisation services and serve as an indirect proxy for service quality.

Lastly, the geographical accessibility of the practices will be proxied using the average distance patients live from their GP. Using the Lower Super Output Area (LSOA)-registered patient population (LSOAs are small statistical areas representing neighbourhoods of ~ 1500 people), we will calculate the straight-line distance from each LSOA centroid to the GP’s physical location. A weighted average of these distances will be applied to each GP based on the number of registered patients for each LSOA. Where feasible, we will explore replacing the straight-line distances with road-based travel times.

Table 2 provides a description of all covariates, their sources and reporting frequency. Linkage to the COVER dataset will be done using a unique identifying code for each GP. For variables reported annually, the value for each year will be applied to all corresponding quarters within that calendar year. Monthly reported covariates will be averaged across each 3-month calendar period to align with the quarterly COVER data. IMD (2019) and ethnicity (2021) are single time point measures and will therefore be applied across all quarters.

**Table 2:** Description, sources and reporting frequency of covariates

Covariate	Description	Data source	Frequency of reporting and data availability
Number of children aged 0–4	Count of children aged 0–4 years out of the total registered population at each practice.	NHS England Digital	Monthly
Proportion of males	Calculated as the percentage of male children aged 0–4 years out of the total registered population at each practice.	NHS England Digital	Monthly
IMD 2019	The IMD is a widely used composite measure of area-level deprivation in England.^14 46 48^ It is calculated using 39 indicators grouped into seven domains: income, employment, health and disability, education and skills, crime, barriers to housing and services and living environment.^49^ These scores are originally calculated at the LSOAs and for GPs, IMD scores are population weighted to reflect the level of deprivation among the registered population.	Fingertips* (Indicator ID:93553)	Single time point (2019)
Proportion of patients by ethnic group	The proportion of black, Asian and mixed ethnicity as reported in the 2021 census: Black (black, black British, black Welsh, Caribbean or African) Asian (Asian, Asian British or Asian Welsh) Mixed (mixed or multiple ethnic groups) Estimates will be derived for each GP by weighting LSOA-level ethnic group proportions (from the 2021 census) according to the number of patients registered from each LSOA, based on April 2021 GP registration data.	Census 2021 (TS021—ethnic group) NHS England Digital (for GP registered population by LSOA)	Single time point (2021)
QOF	The QOF is a national system used to assess how well GPs deliver care across a range of clinical and organisational domains.^50^ Practices are awarded points based on their performance, and financial incentives are tied to the total points achieved. In the Fingertips dataset, QOF performance is reported as the percentage of points attained out of the maximum possible points, with higher values indicating better overall performance.	Fingertips* (Indicator ID:295)	Annually (2010–2023)
Percentage of patients satisfied with their GPs	This measure is derived from the GP annual patients survey. It represents the proportion of respondents who report a positive overall experience with their GP and expressed as percentages of total survey respondents at each practice.	Fingertips* (Indicator ID:93438)	Annually (2018–2024)
Percentage of patients with caring responsibility	This measure is derived from the GP annual patients survey. It represents the proportion of respondents who reported having caring duties for others and expressed as percentages of total survey respondents at each practice.	Fingertips* (Indicator ID:352)	Annually (2012–2024)
Total funding per patient	Total funding per patient reflects the average financial resources available per patient within each PCN. It is calculated by dividing the total funding received by the PCN and its member practices by the combined registered patient population, then applied uniformly to all practices within the PCN. This variable serves as a proxy for the financial capacity of practices to support service delivery.	Fingertips* (Indicator ID:93950)	Annually (2019–2021)
Clinical workforce per 10 000	The PCN-level full time equivalent total is divided by the registered patient population and standardised per 10 000 patients. This average is then applied to all practices within the PCN.	Fingertips* (Indicator ID:93966)	Quarterly by calendar year (2021 Q3 to 2024 Q1)
Average number of appointments per registered patient	Appointments per patient is a 12-month rolling sum of all booked appointments divided by the number of registered patients at the end of that period. The metric reflects overall service use within GPs.	Fingertips* (Indicator ID:93934)	Monthly (October 2022 to March 2024)
Average distance from GP	A proxy measure of geographical accessibility. This variable represents the average straight-line distance between the GP practice and its registered population, calculated using the LSOA centroid of each patient’s area. For each GP, distances from the centroid of each LSOA where patients are registered will be calculated to the GP’s location. A weighted average will then be derived, with weights based on the number of registered children per LSOA.	NHS England Digital (for GP registered population by LSOA) ONS Open Geography Portal (LSOA centroids) ONS Open Geography Portal (GP physical location)	Single time point at the intervention starting point (October 2023)

* National general practice profiles, available via the department of health and social care fingertips platform.

GP, General practice; IMD, English Index of Multiple Deprivation; LSOA, Lower layer Super Output Area (a statistical geographical unit in the UK that represent a small area usually compromising of 1500 residents); NHS, National Health Service; ONS, Office for National Statistics; PCN, primary care network; QOF, Quality and Outcomes Framework.

### Intervention details

The intervention targeted parents and carers of children aged 13 months to 5 years and 11 months who were registered with practices in the central and north Liverpool primary care networks (PCNs), areas known for lower childhood vaccination rates and higher levels of deprivation.

Activities were designed and delivered as part of a community-led, creative health approach. The model adapted standard quality improvement (QI) tools for use in community settings and was implemented through establishing a multidisciplinary Community Innovation Team (CIT) comprising community organisations, volunteers, community champions, creatives, primary care and public health professionals. The team received phased training in QI methodology, beginning with the use of local GP data to identify and target populations with low uptake.

To inform intervention design, the CIT conducted a behavioural insight survey with 116 parents and carers of partially or unvaccinated children aged 1–5 years and consulted local health providers involved in childhood vaccination. This was followed by a structured root cause analysis to identify feasible priority actions that tackle root causes locally. Findings revealed widespread concerns about vaccine safety and efficacy, difficulty in identifying misinformation and reduced trust in health authorities and National Health Service (NHS) information. In response, the CIT co-developed a multicomponent intervention to deliver locally relevant health messaging.

The intervention was implemented between 1 September 2023 and 1 June 2024 across central and northern Liverpool. Activities included community outreach events, school-based coffee mornings and a series of reflective discussions, all of which were designed to increase awareness of the MMR vaccine, build trust and support parents and carers in accessing vaccination services. In total, 22 events were held across nine sites. These were supplemented by digital and printed education material, including targeted MMR-related YouTube video links, ‘Measles Machine’ flyers and reminder text messages sent to nearly 2000 parents of partially or unvaccinated children.

**Figure 1:**
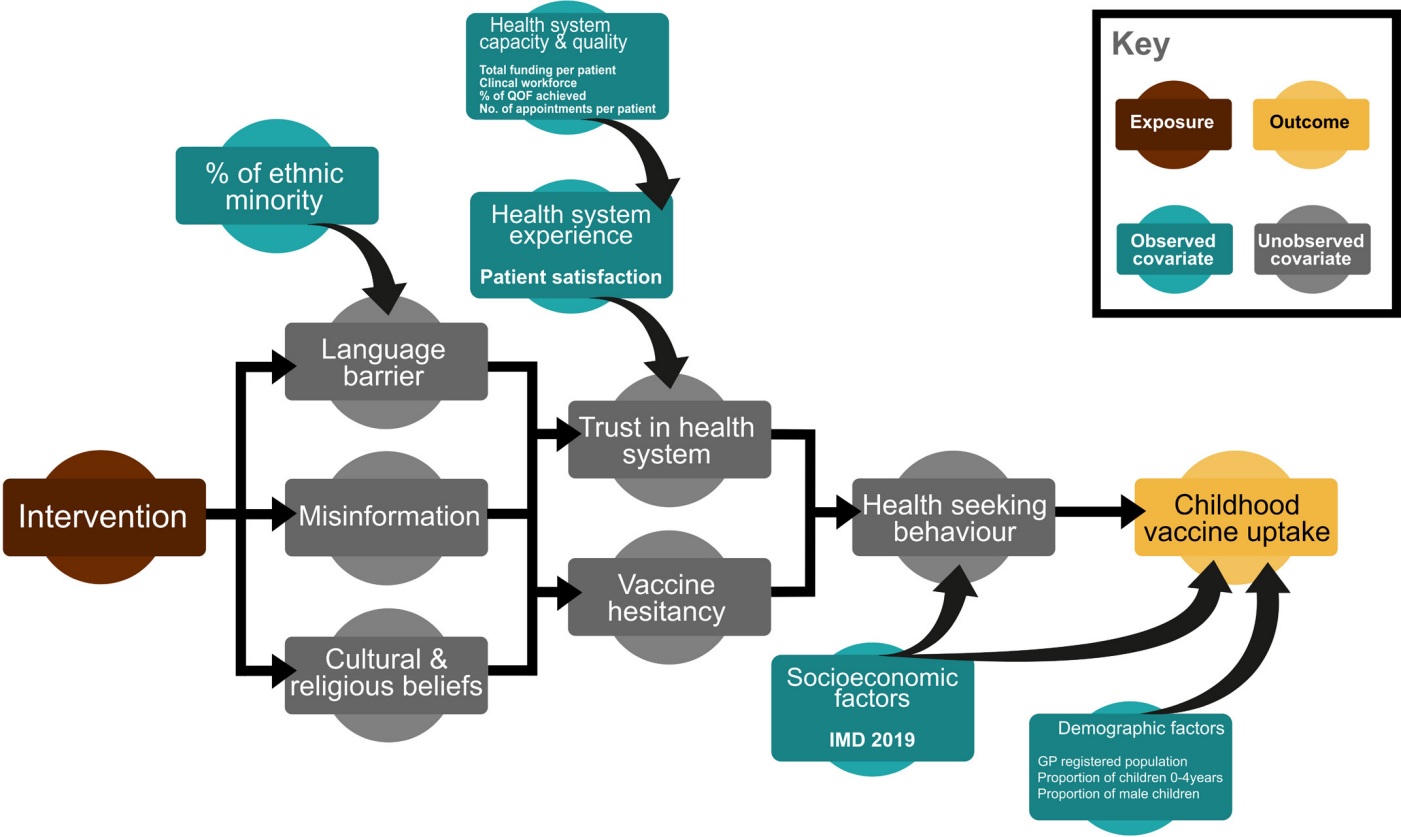
Conceptual diagram that hypothesises the effect of the community-based intervention on the uptake of childhood immunisation.

Informed by the behavioural insights survey findings, we developed a conceptual diagram representing an a priori hypothesis of how the HELP intervention is expected to influence childhood vaccine uptake (figure 1).

### Analysis

We will use the SCM, an observational quasi-experimental approach that extends the traditional difference-in-differences (DiD) framework,^27^ to estimate the impact of the intervention on childhood vaccine uptake. While DiD assumes that the intervention and comparison groups would have followed parallel trends in the absence of the intervention, SCM constructs a weighted combination of untreated units, called a ‘synthetic control’, that mirrors the pre-intervention characteristics and outcome trends (ie, vaccine uptake) of the intervention units.^28^ This allows for a more robust estimation of the counterfactual, particularly when the parallel trends assumption is unlikely to hold. This method has been previously used to evaluate the population-level impact of various public health interventions,^34 35^ including those targeting vaccine uptake,^36 37^ demonstrating its suitability for the current study.

**Figure 2:**
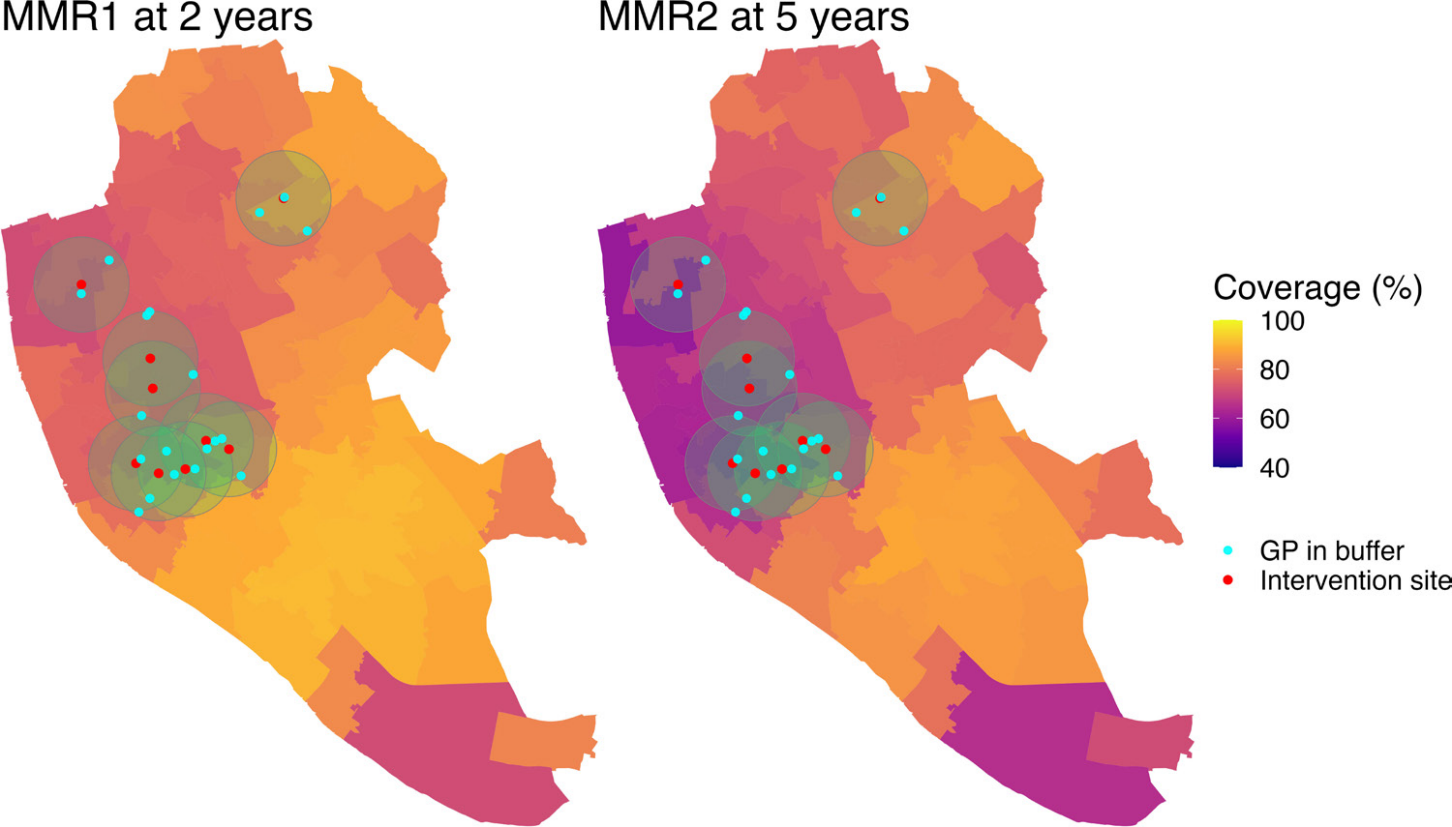
Coverage of MMR1 at 2 years and MMR2 at 5 years across Liverpool by MSOA in the year preceding the intervention (April 2022 to March 2023). Red dots with green circles represent the locations of intervention events with a 1 km surrounding buffer, and cyan dots represent the population-weighted centroid of intervention GPs. Coverage data are based on GP-level uptake reported through the COVER programme. COVER, Cover of Vaccination Evaluated Rapidly; GP, general practice; MMR, measles, mumps and rubella; MSOA, Middle Layer Super Output Area.

GPs will serve as our unit of analysis, with the target population being registered children aged under 5 years who were eligible for routine childhood vaccinations between April 2019 and March 2025, as reported in the COVER dataset. The intervention group is defined as practices whose population-weighted centroids fell within a 1 km radius of any of the nine intervention sites. As such, 19 practices will be included in the intervention group (see figure 2), with all remaining practices in England forming the donor pool for constructing the synthetic control group. We will apply SCM for microdata,^38^ which is specifically suited to settings involving numerous small scale units, such as GPs. Matching will be performed using calibration weights for the pre-intervention uptake of included vaccines and the previously mentioned covariates. The final inclusion of covariates in the SCM will also be guided by their practical contribution to achieving balance between intervention and control units.

The pre-intervention period spans Q1 to Q18 (April 2019 to September 2023), with Q19 to Q24 (October 2023 to March 2025) defining the post-intervention period. The average treatment effect will be estimated as the average difference in vaccine uptake between the intervention and synthetic control groups in the post-intervention period.

The statistical significance of estimated effects will be assessed using the permutation inference, following the method described by Robbins and Davenport.^39^ We will perform 250 permutations, reassigning treatment status to control units to generate a distribution of treatment effects under the null hypothesis (ie, no difference in vaccine uptake between the intervention and control groups). All analyses will be conducted using R V.4.4.2 and the microsynth package.^39^

### Sensitivity analysis

Given the community-based nature of the intervention, there is potential for spillover effects beyond the identified intervention practices. To mitigate this effect, we will conduct a sensitivity analysis excluding other non-intervention GPs located within the central and north Liverpool PCNs from the donor pool practices used to construct the synthetic control group. We will also incorporate a one-quarter lag at the start of the post-intervention period (ie, starts at Q20 instead of Q19) to allow sufficient time for any changes in vaccine uptake to become observable.

To assess the sensitivity of the estimated effect to the exposure’s spatial definition, we will test alternative buffer distances (0.5 km, 1.5 km and 2 km) from intervention event sites. We will further attempt to exclude practices that may have been affected by other concurrent vaccine-related initiatives implemented elsewhere in England. To identify such initiatives, an audit of interventions form will be distributed to immunisation leads at the UK Health Security Agency and to public health leads across the nine local authorities in Cheshire and Merseyside via the Champs Public Health Collaborative. A copy of the form is provided in online supplemental material 1.

Further sensitivity analyses will involve adjusting the pre-intervention period to begin after the final phase of national COVID-19 restrictions in England (February 2022 to Q12 in our dataset), as it could bias the counterfactual estimation. For example, tiered restrictions during the pandemic varied in stringency across England. This has led to regional variation in childhood vaccine uptake^40^ and will consequently affect the SCM matching.

In addition, we will rerun the evaluation using vaccine uptake aggregated to a small area neighbourhood level (LSOA), which may better reflect the community-based nature of the intervention. Finally, to test the robustness of our findings, we will explore alternative approaches to constructing synthetic controls. These may include methods that incorporate negative control series or rely on different assumptions about the counterfactual.

### Timeline

The study uses data from the COVER programme, with data acquisition limited to downloading and curating publicly released datasets. The audit form is planned for January to April 2026. Data analysis will begin following completion of the audit, and the study is expected to conclude by January 2027.

### Patient and public involvement

Although there were no patients or members of the public directly involved in the design of this evaluation, the intervention itself was co-produced with members of the public, as described in the intervention details section. Also, as part of the ReCITE project, a co-researcher training programme has been developed to support public involvement throughout the research process. This programme provides training in research methods for members of the public who have either participated in or been affected by the intervention. Trained co-researchers will contribute to the interpretation of results and the communication of findings to lay audiences.

### Ethics and dissemination

This study will be conducted as part of the ReCITE project, which has received ethical approval from the Liverpool School of Tropical Medicine Research Ethics Committee (Reference: 24–018). Findings will be shared with the project funder, submitted for publication in a peer-reviewed journal and communicated to the public through plain language summaries. The analytical code will be made publicly available through an online repository (GitHub), along with any processed data derived from open-access sources. This will enable full reproduction of the analysis and support methodological reuse in similar evaluations.

## Discussion

### Summary

This protocol outlines the design of a population-level evaluation of a community-led initiative aimed at increasing childhood vaccine uptake in Liverpool using publicly available GP-level data and SCM. While the primary outcomes are MMR1 and MMR2 uptake, the inclusion of other childhood vaccines, such as the 6-in-1, rotavirus and PCV booster, in the analysis allows for the assessment of broader shifts in routine immunisation behaviour, which may also be influenced by community engagement activities. This study also serves as a methodological pilot for evaluating similar hyper-localised community-led interventions targeting vaccination uptake in the UK, including interventions implemented under the ReCITE programme (Building Research by Communities to Address Inequities Through Expression),^41^ which tackles health inequities across the Cheshire and Merseyside region in the UK.

### Expected results

The decline in childhood vaccine uptake has been a longstanding issue in the UK, possibly initially influenced by the introduction of the government’s austerity measures in 2012.^42^ This was further exacerbated by changes to the financing structure of childhood immunisation services in 2015,^43^ resulting in the fragmentation of services across commissioning bodies (NHS, local authorities).^44^ A key consequence was the reduction in the responsibilities of health visitors, despite their prominent role as a trusted communication channel for families.^45^ Considering the design of the HELP multi-component intervention, with a particular emphasis on tailored health messaging and local engagement through dialogue, we hypothesise that it could help rebuild trust and establish new channels of communication with parents and caregivers, ultimately contributing to improving vaccine uptake.

Previous studies of multicomponent interventions incorporating education, reminders and provider engagement have demonstrated average improvements of approximately 25% in vaccine uptake under trial conditions.^21^ However, such effects may be attenuated in population-level evaluations. Therefore, we anticipate a relative increase in the uptake ranging from 5 to 15 percentage points compared with the synthetic control group. The magnitude of the effect will also depend on local engagement and the potential lag between exposure to the intervention and the behavioural response. Nonetheless, given the persistently low baseline coverage, even modest improvements in uptake may represent meaningful public health gains.

### Limitations

First, although we will attempt to account for confounding during the construction of the SCM, bias due to unobserved characteristics remains a possibility as with all other observation studies. Not all relevant determinants of vaccine uptake, such as parental attitudes, language proficiency or local outreach efforts, can be accounted for using open access data aggregated data. Additionally, some covariates (eg, IMD and ethnicity) are available only as single point measures. Although area-level deprivation tends to remain stable over time,^46^ the use of fixed values may not fully capture recent demographic shifts. Nonetheless, these indicators remain important proxies for structural disadvantage, which is known to shape immunisation behaviour.

Second, the representativeness of variables from the national GP patients survey might be limited due to the low response rate of around 27%.^47^ Responses might reflect experiences of those who are already more engaged with healthcare services, while under-representing the populations that are less likely to participate. Still, these variables remain valuable for capturing the broader patterns of patient engagement across GPs.

Third, there is currently no centralised registry or reporting system in England that documents all vaccination equity improvement initiatives and the degree to which they are community-led. Our efforts to identify other similar vaccination initiatives through a brief audit might not comprehensively map every relevant intervention that may have been active during the study period. However, this is a broader challenge that affects all evaluations of localised public health interventions, particularly those of non-randomised design.

Finally, our decision to use GP level aggregated data might affect SCM matching due to the lack of granularity in covariate and outcome measurements. Without access to individual-level records, it is impossible to account for the within-practice variation. While a similar evaluation could be replicated using individual-level records, access to such data is often restricted. Thus, by testing the feasibility of using open access sources such as the COVER dataset, we aim to demonstrate a reproducible approach that can be adopted in local settings with limited access to line-listing data. In doing so, we also will inform the minimum data requirements for similar evaluations and provide reusable analytical code to support replication.

## Conclusion

In conclusion, this study will be among the first to apply SCM to evaluate the population-level impact of a community-led intervention on uptake of five childhood vaccines in the UK. It will offer policy relevant evidence on the effectiveness of hyper-localised engagement strategies in increasing immunisation coverage among preschool children.

## Supplementary material

10.1136/bmjopen-2025-111500 online supplemental file 1

## Data Availability

Data are available in a public, open access repository.
