# Supplementary material for: Impact of a community-led intervention on the uptake of childhood vaccines in Liverpool: a protocol for a synthetic control evaluation
Source: BMJ Open. 2026 Jan 21;16(1):e111500. doi: 10.1136/bmjopen-2025-111500 (PMC12829374; doi:10.1136/bmjopen-2025-111500)
Supplement: online supplemental file 1 [file bmjopen-16-1-s001.pdf]

# Mapping Childhood Vaccination Initiatives in England (2021-2025)

Dear colleague,

We are conducting an evaluation of a community-led childhood vaccination initiative (the Health Equity Liverpool Project [HELP]), which aimed to improve childhood vaccine uptake through locally delivered outreach and engagement activities across Liverpool, England. This evaluation forms part of the ReCITE project, funded by the Arts and Humanities Research Council (project number: AH/Z505341/1), and is ethically approved by the Liverpool School of Tropical Medicine (reference: 24-018).

To ensure the accuracy of our findings, we are collecting information on any other childhood vaccination-related initiatives that were implemented locally (post-COVID) between January 2021 and March 2025. We would greatly appreciate your help in identifying any such programmes or campaigns that may have taken place in your area during this time. **Please note that general awareness campaigns (e.g. social media posts not tied to a specific geographic location or community engagement effort) are not within the scope of this form.**

**The survey should take no more than 5 minutes to complete.**

If you have any questions about this survey, please contact: Mohammed Sherif Amin Email: sherif.amin@liverpool.ac.uk
For further information about the HELP project, please visit: https://www.lstmed.ac.uk/news-events/blogs/recite-final-help-report

## Respondent Information

**Organisation/Local authority** *

**Role or Job Title** *

## Intervention Details

### Geographic Area *

Which areas did your vaccination-related intervention(s) cover?

- ◯ Liverpool
- ◯ Knowsley
- ◯ Sefton
- ◯ Wirral
- ◯ St Helens
- ◯ Halton
- ◯ Cheshire East
- ◯ Cheshire West and Chester
- ◯ Warrington
- ◯ Other

### Intervention Active period *

Were any vaccination-related programmes or interventions targeting children aged 0–5 implemented in your area between January 2021 and March 2025?

- ◯ Yes
- ◯ No

### Delivery Period (Start date) *

What was the start date of the intervention delivered?

### Delivery Period (End date)

What was the end date of the intervention delivered? **(If the intervention is still running, please ignore this question).**

**Target population** *

Who was the primary target of the intervention(s)?

- [ ] Children under 5 years
- [ ] Parents/carers
- [ ] Schools or nurseries staff
- [ ] Healthcare providers
- [ ] Other

Please list any specific sites targeted by your intervention (e.g., schools, nurseries, general practices, community venues, etc.), including names and postcodes. *

If the intervention was deployed more broadly (i.e., local authority level), please specify the local authorities involved.

Did the intervention target specific childhood vaccine(s)? *

- ◯ **Yes**, the intervention focused primarily on certain childhood vaccine(s)
- ◯ **No**, the intervention aimed to improve childhood vaccination more broadly, without focusing on a specific vaccine
- ◯ Other

Please list the targeted vaccine(s)? *

Evaluation or data collection *

Was the intervention evaluated, or does it have publicly available data?

- ◯ Yes
- ◯ No
- ◯ Don't know
- ◯ Other

Please describe the intervention(s), including: **1-** Key activities (e.g., school events, mobile vaccination units, communications campaigns, etc.). **2-** Number of activities/events held. **3-** Geographic area(s) covered (e.g., schools, nurseries, general practices, neighbourhoods, etc.). **4-** Any other information you think might be useful. *

This content is neither created nor endorsed by Microsoft. The data you submit will be sent to the form owner.

Microsoft Forms
