# Supplementary material for: Impact of a community-led intervention on the uptake of childhood vaccines in Liverpool: a protocol for a synthetic control evaluation
Source: BMJ Open. 2026 Jan 21;16(1):e111500. doi: 10.1136/bmjopen-2025-111500 (PMC12829374; doi:10.1136/bmjopen-2025-111500)
Supplement: Supplementary file 2 [file bmjopen-2025-111500.reviewer_comments.pdf]

PEER REVIEW HISTORY

BMJ Open publishes all reviews undertaken for accepted manuscripts. Reviewers are asked to complete a checklist review form and are provided with free text boxes to elaborate on their assessment. These free text comments are reproduced below.

ARTICLE DETAILS

Title (Provisional)

Impact of a community-led intervention on the uptake of childhood vaccines in Liverpool: A protocol for a synthetic control evaluation

Authors

Amin, Mohammed Sherif; Zhang, Xingna; Green, Mark Alan; Holford, Dawn; Hemingway, Charlotte; Ismail, Amina; Moran, James; Doyle, Vicki; Taylor, Cait; Taegtmeyer, Miriam; Hungerford, Daniel

---

VERSION 1 - REVIEW

**Reviewer**	**1**
**Name**	**Tung, Tao-Hsin**
**Affiliation**	**Taizhou Hospital of Zhejiang Province, Evidence-based Medicine Center**
**Date**	**17-Dec-2025**
**COI**	**None**

The study design and methods employed are generally appropriate. However, there are several areas where the protocol requires strengthening.

1. Please provide additional details about the target population. Specifically, clarify who constitutes the target population.

2. Describe the measures taken to prevent various sources of bias, including sequence generation (to address selection bias), allocation sequence concealment (also to mitigate selection bias), blinding of participants and personnel (to reduce performance bias), blinding of outcome assessment (to minimize detection bias), handling of incomplete outcome data (to address attrition bias), prevention of selective outcome reporting (to avoid reporting bias), and other potential biases.

3. The authors should include remarks on selection bias in the protocol within the perceived limitations subsection.

Overall, congratulations to the authors on their enthusiasm for this trial. Nevertheless, the protocol does not meet the required quality standards for publication in BMJ Open without revisions.

VERSION 1 - AUTHOR RESPONSE

Reviewer 1

Comment 1
Please provide additional details about the target population. Specifically, clarify who constitutes the target population.

Response:
Thank you for this comment. We have further clarified the target population in the Methods section in the revised manuscript, lines 287-289: "*GPs will serve as our unit of analysis, with the target population being registered children aged under five years who were eligible for routine childhood vaccinations between April 2019 and March 2025, as reported in the COVER dataset.*"

Comment 2
Describe the measures taken to prevent various sources of bias, including sequence generation (to address selection bias), allocation sequence concealment (also to mitigate selection bias), blinding of participants and personnel (to reduce performance bias), blinding of outcome assessment (to minimize detection bias), handling of incomplete outcome data (to address attrition bias), prevention of selective outcome reporting (to avoid reporting bias), and other potential biases.

Response:
This study uses a synthetic control analysis framework [1], a quasi-experimental observational design, applied to the routinely collected open-access COVER data, as described in the Introduction (lines 165-166: "*We aim to apply the SCM to evaluate the impact of the HELP project on the uptake of five childhood vaccines*") and Methods and Analysis sections (172-175: "*This study will utilise publicly available data from the Cover of Vaccination Evaluated Rapidly (COVER) programme [30], which provides quarterly statistics on the uptake of routine childhood immunisations for children aged five years and under at the general practice (GP) level in England.*"). We have also justified this design choice in the Introduction by explaining why a controlled trial was not feasible for evaluating this complex, community-based intervention (lines 152-155: "*While randomised controlled trials remain the gold standard, they are often unfeasible for evaluating complex health system interventions involving numerous interacting components and stakeholders within a dynamic social context.*").

We have now further clarified the study's observational design in the Analysis section of the revised manuscript (lines 275-276): "*We will use synthetic control methods, an observational quasi-experimental approach that extends the traditional difference-in-differences (DiD) framework*". We have also revised the Limitations section to explicitly attribute the risk of unobserved confounding to the study's observational nature (lines 402-403: "*bias due to unobserved characteristics remains a possibility as with all other observation studies.*").

Given the quasi-experimental, observational nature of the study, the intervention was not assigned by the investigators; rather, it was implemented through a community-led approach, as mentioned in lines 245-251. **Consequently, trial-specific procedures such as sequence generation, allocation**

sequence concealment, and blinding of participants, personnel, or outcome assessors are not applicable to this study design.

Comment 3
The authors should include remarks on selection bias in the protocol within the perceived limitations subsection.

Response
As discussed in our response to Comment 2, this study is an observational evaluation in which the intervention was not assigned by the investigators. As such, selection bias arising from participant recruitment or allocation does not apply in the same way as in randomised studies.
